# Supplementary material for: Consore: A Powerful Federated Data Mining Tool Driving a French Research Network to Accelerate Cancer Research
Source: Int J Environ Res Public Health. 2024 Feb 7;21(2):189. doi: 10.3390/ijerph21020189 (PMC10887639; doi:10.3390/ijerph21020189)
Supplement: Supplementary file 1 [file ijerph-21-00189-s001.zip › ijerph-2748214-supplementary.pdf]

International Journal of
Environmental Research and Public Health

MDPI

Table S1: Comparison between the 3 main french data warehouse solutions

	Consore	Dr. Warehouse	eHop
**Field**	Oncology	General medicine / rare disease	General medicine
**Scope**	11 comprehensive cancer centers (CLCC)	some university hospitals + one CLCC	several university hospitals + one CLCC
**NLP embedded**	yes	yes	yes
**Type of storage**	No-SQL (ElasticSearch)	RDBMS (Oracle)	RDBMS (Oracle)
**Interconnected network**	yes	no	no
**Open-source code**	no	yes	no

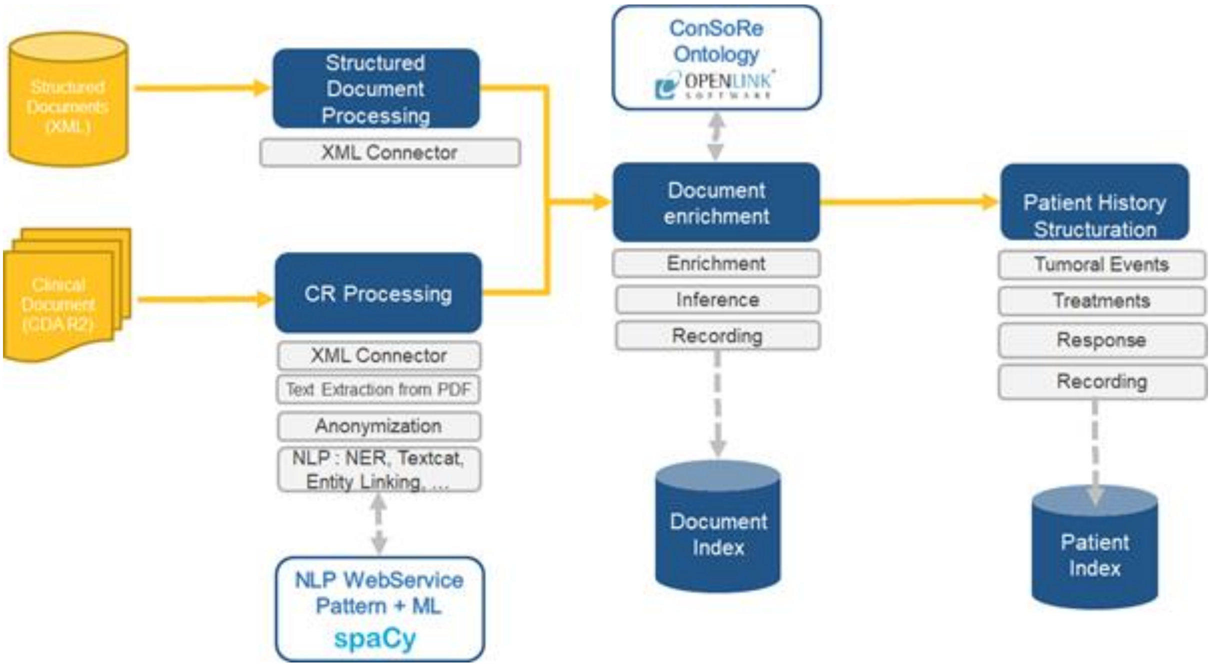

Figure S1: Architecture that integrates advanced NLP techniques with probabilistic models and machine learning algorithms.
